# Recommendations for Multicentre Clinical Trials Involving Dosimetry for Molecular Radiotherapy

**DOI:** 10.1016/j.clon.2020.12.002

**Published:** 2021-02

**Authors:** J. Taprogge, J. Wadsley, E. Miles, G.D. Flux

**Affiliations:** ∗ National Radiotherapy Trials Quality Assurance (RTTQA) Group, Joint Department of Physics, Royal Marsden NHSFT, Sutton, UK; † The Institute of Cancer Research, London, UK; ‡ Weston Park Hospital, Sheffield, UK; § National Radiotherapy Trials Quality Assurance (RTTQA) Group, Mount Vernon Cancer Centre, East and North Hertfordshire NHS Trust, Northwood, UK; ¶ Joint Department of Physics, Royal Marsden NHSFT, Sutton, UK

**Keywords:** Dosimetry, molecular radiotherapy, multicentre trial, quality assurance

## Abstract

Multicentre clinical trials involving a dosimetry component are becoming more prevalent in molecular radiotherapy and are essential to generate the evidence to support individualised approaches to treatment planning and to ensure that sufficient patients are recruited to achieve the statistical significance required. Quality assurance programmes should be considered to support the standardisation required to achieve meaningful results. Trials should be designed to ensure that dosimetry results from image acquisition systems across centres are comparable by incorporating steps to standardise the methodologies used for the quantification of images and dosimetry. Furthermore, it is essential to assess the expertise and resources available at each participating site prior to trial commencement. A quality assurance plan should be drawn up and training provided if necessary. Standardisation of quantification and dosimetry methodologies used in a trial are essential to ensure that results from different centres may be collated. In addition, appropriate uncertainty analysis should be carried out to correct for differences in methodologies between centres. Recommendations are provided to support dosimetry studies based on the experience of several previous and ongoing multicentre trials.

## Highlights

• Multicentre clinical trials involving a dosimetry component are becoming more prevalent in molecular radiotherapy.
• Recommendations are provided to support molecular radiotherapy dosimetry studies based on experience of previous/ongoing multicentre trials.
• In the future, the use of global calibration factors will potentially allow for expansion of existing imaging networks.

## Introduction

Most molecular radiotherapy (MRT) treatments are given with a fixed activity administration of radioisotope, accepting that this will lead to a wide range of absorbed doses delivered both to tumours and to organs at risk. Patient dosimetry is seldom carried out either to predict or verify the radiation doses delivered. This is in marked contrast to external beam radiotherapy (EBRT) and brachytherapy [1]. As MRT becomes recognised as a form of systemic radiotherapy rather than conventional chemotherapy, the prospect of personalised treatment planning and optimisation based on patient dosimetry must be considered. Due to radiobiological factors, in particular the range of radiation emissions, relative biological effectiveness, heterogeneous dose distribution [2] and dose rate effects [3], absorbed doses delivered from MRT cannot be directly correlated to, for example, the absorbed dose delivered from a 2 Gy per fraction course of EBRT. Treatment regimens therefore cannot be readily adapted from conventional protocols used for EBRT. Single centres are seldom able to recruit sufficient patients to achieve the statistical significance required to report on study end points [4,5]. Large prospective, randomised, multicentre studies are therefore required to show the value of personalised treatment planning in MRT [6].

## The Evidence for Dosimetry

The potential for dosimetry-based treatment planning has been shown for many therapy procedures [6]. Correlations between absorbed doses and clinical outcomes following MRT have been reported for several radiopharmaceuticals in single-centre clinical studies, aided by the wide range of absorbed doses delivered [7]. Dewaraja *et al.* [8] found that mean tumour absorbed doses correlated with improved progression-free survival after ^131^I-tositumomab radioimmunotherapy. Wierts *et al.* [9] used pre-therapy ^124^I positron emission tomography/computed tomography (PET/CT) lesion dosimetry in thyroid cancer patients treated with a fixed activity of ^131^I-NaI and observed a dose–response relationship for thyroid remnants and metastases. A correlation of absorbed dose with successful ablation was also shown for thyroid cancer treated with radioiodine [10]. Ilan *et al.* [11] found a significant correlation between tumour absorbed doses and tumour shrinkage for pancreatic neuroendocrine tumours treated with ^177^Lu-DOTATATE. Violet *et al.* [12] observed a significant correlation between whole-body tumour dose and prostate-specific antigen response in metastatic castration-resistant prostate cancer patients treated with ^177^Lu-PSMA-617. Barone *et al.* [13] showed that kidney toxicity after ^90^Y-DOTATOC therapy is absorbed dose dependent.

## Multicentre Clinical Trials Incorporating Dosimetry

Although a large number of clinical trials for new and established radiopharmaceuticals have implemented dosimetry, a recent survey found that MRT practice across Europe varies significantly, especially with respect to the implementation of personalised treatment based on dosimetry [1]. Collation of results from multiple centres in MRT dosimetry trials requires standardised quantitative single photon emission computed tomography(/computed tomography) SPECT(/CT) acquisitions [14].

In recent years, significant efforts have been made to improve quantification for gamma camera imaging [[15], [16], [17]] and work has started on the standardisation of quantification in MRT. Zimmerman *et al.* [18] carried out an international comparison of the activity measurement of ^177^Lu. In a later study, they evaluated the accuracy and reproducibility of activity quantification of planar and SPECT imaging in a multicentre setting with an IAEA phantom study for ^133^Ba, which was used as a surrogate for ^131^I [19]. Peters *et al.* [20] carried out phantom measurements as part of a multivendor and multicentre study to assess the quantitative accuracy and inter-system variability of SPECT/CT systems. Both Zimmerman *et al.* [[18], [19]] and Peters *et al.* [20] found that absolute SPECT quantification in a multicentre, multinational setting is feasible, but that standardisation of image acquisition, reconstruction parameters and processing is key. Wevrett *et al.* [21] carried out an international inter-comparison exercise for quantitative imaging of ^177^Lu to investigate consistency between clinical sites. Gregory *et al.* [22] and Taprogge *et al.* [23] established networks of centres able to carry out standardised radioiodine activity quantification.

Hänscheid *et al*. [24] carried out an international, prospective, controlled, randomised study of radioiodine ablation for differentiated thyroid cancer to compare stimulation with recombinant human thyroid stimulating hormone and thyroid hormone withdrawal. Standardised acquisition and processing protocols were used and dosimetry results calculated at a central dosimetry hub. Sündlov *et al.* [25] carried out a phase II, multicentre, prospective clinical trial using ^177^Lu-DOTATATE to treat metastatic neuroendocrine tumours in two centres in Sweden.

Recent examples of multicentre MRT clinical trials that involved standardisation of the acquisition and reconstruction parameters with centralised dosimetry are SEL-I-METRY [22,26,27] and MEDIRAD [23]. SEL-I-METRY (EudraCT no. 2015-002269-47) is a phase II clinical trial to investigate the potential of selumetinib in resensitising patients with advanced iodine refractory differentiated thyroid cancer to radioiodine. Uniquely, the SEL-I-METRY trial implemented a quality assurance programme in association with the UK Radiotherapy Trials Quality Assurance (RTTQA) Group to achieve standardisation across the centres in the trial. MEDIRAD is a European Commission Horizon 2020-funded project. Work package 3 (WP3) within MEDIRAD aims to measure the range of absorbed doses delivered to healthy organs from radioiodine ablation of thyroid cancer. A linked prospective observational study in the UK (INSPIRE, NCT04391244) is at the stage of initial recruitment and currently under development to allow multicentre participation.

## Recommendations for Molecular Radiotherapy Dosimetry Multicentre Clinical Trials

Multicentre dosimetry trials require careful planning to ensure that data can be collected, stored and directly compared. Standardisation of image quantification and dosimetry methods with appropriate uncertainty analysis is essential. Systematic or random errors in the quantification, outlining and dosimetry calculations due to imperfect equipment calibrations and differences in processing of data may lead to large uncertainties in the calculated absorbed doses [28]. This could potentially result in a dose–response relationship not being detected or a bias of the data used for the analysis of end points of the clinical trial. Data transfer and storage must be set up to ensure that essential information stored in DICOM tags and non-DICOM data are available for dosimetry processing and review of data as part of a centralised quality assurance programme. The following recommendations are provided to facilitate the successful preparation and running of multicentre MRT studies that incorporate dosimetry, based on the experience of the multicentre SEL-I-METRY and MEDIRAD trials in the UK and mainland Europe. These recommendations are generated with the guidance and extensive experience of radiotherapy quality assurance for multinational EBRT trials.

### Trial Quality Assurance Programme

Recommendation 1: MRT clinical trials involving a component of dosimetry should incorporate a clinical trials quality assurance programme similar to that in place for EBRT.

Clinical trials quality assurance programmes help to ensure that trial data are collected and documented following the trial protocol, good clinical practice and other relevant guidance [[29], [30], [31]]. For MRT clinical trials involving dosimetry, the trial quality assurance should consist of a set of planned, systematic activities to minimise bias due to variations in the dosimetry results from different centres. This should include a site set-up or facility questionnaire and standard operating procedures (SOPs) for site set-up and dosimetry calculations [32]. Examples of site set-up SOPs were published by Gregory *et al.* [22] in the supplementary material. Furthermore, trial monitoring activities should be defined to ensure adherence to trial protocols at all stages throughout the clinical trial. Protocol deviations from SOPs can be minimised by a case-by-case check of the imaging and reconstruction parameters at a central dosimetry hub [33].

Although clinical trials quality assurance programmes are routine practice in EBRT [34,35], through the Global Quality Assurance of Radiation Therapy Clinical Trials Harmonization Group, RTQA [36] and the Radiotherapy Trials Quality Assurance (RTTQA) Group in the UK, further work is required to implement similar quality assurance programmes in all MRT dosimetry clinical trials. MRT can benefit from the experience gathered in EBRT regarding the set-up and running of multicentre clinical trials involving dosimetry.

### Site Set-up/Facility Questionnaires

Recommendation 2: Communication should be facilitated between key staff at each centre to promote sharing of experience and resources.

The expertise and resources available at each centre, including medical physics support, experience with MRT dosimetry, gamma camera availability and ancillary equipment required (i.e. radionuclide calibrators) should be assessed with site set-up/facility questionnaires. The questionnaire should identify key local personnel/staff in the multiprofessional team, including a named medical physicist to assist with site set-up measurements, quality control procedures and data handling. All key local personnel/staff should be informed about progress of essential stages in the set-up and running of the clinical trial through regular communications and conference calls. The responses from the questionnaire will identify centres or individuals that may require further training or support for the site set-up measurements at each centre.

### Standardisation of Imaging Acquisition Protocols

Recommendation 3: Image acquisition and dosimetry protocols should be standardised as far as reasonably practicable allowing for differences in local availability of resources such as SPECT or SPECT/CT systems.

Absorbed dose calculations require serial imaging over several days following therapy. Patients may therefore be asked to make multiple return visits to hospital for further imaging, which can also have resource implications for nuclear medicine departments in terms of both staff and equipment time. In contrast to EBRT, dosimetry procedures are currently often not reimbursed. The requirement for significant additional imaging can therefore increase the costs of academic trials. These factors stress the need for high-quality studies to justify the additional resource requirements to acquire dosimetric information.

### Standardisation of Quantitative SPECT in a Multicentre Setting

Recommendation 4: Gamma camera calibration methodologies and image acquisition and reconstruction protocols should be standardised across clinical trials.

Site set-up measurements are essential for clinical studies involving quantitative imaging. These may include system volume sensitivity calibrations, partial volume corrections and dead-time characterisation. System volume sensitivity is defined as the system's count-rate for a uniform concentration of activity. SPECT recovery coefficients are necessary to correct the observed activity concentration in tomographic imaging for partial-volume and resolution effects [37]. Dead-time factors are applied to correct the observed count-rate of the system for count losses due to detector paralysis at high imaged activity levels.

Experience from multicentre MRT clinical trials [22,23] have shown that such measurements may need to be adapted locally based on radiation protection guidance in different countries and centres. It is essential to ensure that the complexity and time required for such measurements are adapted as necessary, particularly for centres with limited resources.

National metrology institutes play a key role in EBRT to ensure delivery of accurate absorbed doses. Accurate activity measurements are essential for MRT absorbed dose calculations. Traceability of activity measurements is currently not an essential requirement in many countries, but will play an important role to achieve comparable dosimetry results from different centres [14].

### Logistics of Data Transfer

Recommendations 5: Appropriate data transfer facilities for both image DICOM data and associated non-DICOM data collected on case report forms should be established and validated before the clinical trial commences.

DICOM data from the serial imaging of patients and associated non-DICOM data, including injected activities and injection times and dates, will potentially have to be transferred from the participating centres to a centralised dosimetry hub for the dosimetry calculations. Validation of imaging DICOM and associated non-DICOM data transfer before the trial starts is an essential requirement. Possible options for the transfer of DICOM data are image databases and informatics software platforms, such as KHEOPS [38] or XNAT [39], or the use of file sharing services approved for such data transfer. Long-term availability and support of such a service must be ensured.

Patient data must be pseudoanonymised and data encryption should be ensured prior to data upload to these services subject to the respective data protection regulations, such as the General Data Protection Regulation (GDPR). DICOM tags required for the dosimetry processing may be subject to deletion as part of the pseudoanonymisation process. Tests should be included in the data transfer validation to identify missing DICOM tags. Furthermore, DICOM tags for injection times and administered activities are often not populated.

Data transfer methods of non-DICOM data, including case report forms, must be agreed upon prior to the start of the trial to ensure that data missing in the DICOM tags are available at the centralised dosimetry hub.

### Dosimetry Calculations and Collation of Results

Recommendations 6: Dosimetry methodologies including uncertainty analysis should either be standardised across centres or carried out at a central dosimetry hub.

For a multicentre clinical trial, dosimetry calculations may be carried out at a centralised dosimetry hub or at the individual local centre. Local data processing requires strict standardisation and appropriate uncertainty estimation [40] of all steps involved in the dosimetry calculations to allow for results to be compared. A central dosimetry hub can help to reduce the risk of bias that may be introduced when data are processed locally. This risk may be mitigated if local dosimetry centres follow common SOPs for all steps involved in the dosimetry calculations. Dosimetry data should in any case be centrally reviewed following the quality assurance procedures drawn up at the beginning of the study. Local data processing and/or dosimetry calculations can potentially reduce the workload at the central dosimetry or quality assurance hub.

Uncertainty analysis is particularly important in MRT because of the current lack of standardisation and the large uncertainties involved in the image processing steps due to outlining and quantification. Dosimetry methodologies must be agreed upon and if different software packages are used, validation should be carried out to ensure that results can be compared. Commercially available software packages are increasingly available, although software developed in-house may be required, based on the dosimetry application. Quality assurance on the different systems should be carried out to provide evidence that the outputs are comparable.

An essential step in dosimetry calculations is often the outlining of lesions and organs-at-risk. Studies have shown that the inter-operator variability of volume delineation can have a significant impact on the absorbed dose calculations [13,41] and, therefore, the ability to identify dose–response relationships if that is a trial end point.

## Future Directions

Initial studies have shown that inter-system variability for a given vendor and camera type is low if acquisition and reconstruction protocols are standardised across centres so that it may be possible to use the same calibration and correction factors [19,20,22,23]. System parameters including sensitivity, partial-volume effect and dead-time correction could be measured on a number of systems for each vendor and camera type to establish a quantitative imaging database for gamma cameras. This would allow for widespread expansion of the existing imaging network without the requirement of complex site set-up measurements. Further measurements are required in large-scale multicentre settings to verify those initial results.

Nevertheless, the use of global calibration factors from a database of calibration measurements would require centre validation measurements to ensure that results from different centres can be combined and to test the full-dosimetry chain [4,42].

## Conclusions

Large-scale multicentre clinical trials are essential to investigate the potential for personalised treatment planning in MRT. Trials require careful planning to ensure that end points of the trial can be achieved. To encourage patient participation, optimised and accurate dosimetry protocols must be established. Expertise and resources at participating sites must be evaluated and training provided if necessary. Standardisation of quantification and dosimetry together with appropriate uncertainty analysis are key to allow for collation of results across multiple centres. These steps will facilitate the development of the networks required to develop personalised treatment planning for MRT, as is routine for EBRT and brachytherapy.

## Conflicts of Interest

J. Taprogge and G.D. Flux report grants from the Euratom research and training programme 2014–2018 and the National Institute for Health Research (NIHR) and funding from the National Health Service to the NIHR Biomedical Research Centre at The Royal Marsden and the Institute of Cancer Research and NIHR Royal Marsden Clinical Research Facility during the conduct of the study. J. Wadsley reports grants and personal fees from AstraZeneca, grants and personal fees from Sanofi-Genzyme, during the conduct of the study; personal fees from AAA, personal fees from Eisai, personal fees from Lilly, personal fees from Roche, personal fees from Celgene, personal fees from Novartis, personal fees from Ipsen, outside the submitted work.
